# HCMV-encoded miR-UL112-3p promotes glioblastoma progression via tumour suppressor candidate 3

**DOI:** 10.1038/srep44705

**Published:** 2017-03-17

**Authors:** Qing Liang, Kejia Wang, Bin Wang, Qiliang Cai

**Affiliations:** 1 MOE&MOH Key Laboratory of Medical Molecular Virology, School of Basic Medical Sciences, Shanghai Medical College, Fudan University, Shanghai 200032, China; 2 Department of Special Medicine, Qingdao University, Qingdao 266071, Shandong, China

## Abstract

Glioblastoma (GBM) is the most prevalent and lethal type of primary malignant brain tumour. Recent studies suggest that the discovery of human cytomegalovirus (HCMV)-encoded microRNAs (miRNAs) might play a role in the pathogenesis of diseases, including GBM. In this study, we aimed to analyse the expression and function of HCMV-encoded miRNAs in GBM. We found that miR-UL112-3p expression was significantly elevated in GBM, and its expression levels were highly associated with glioma size, differentiation, WHO stage and the overall and disease-free survival of patients. The overexpression of miR-UL112-3p in the GBM cells promoted cell proliferation, clone formation, migration and invasion. In contrast, the down-regulation of miR-UL112-3p exerted an inverse effects. Tumour suppressor candidate 3 (TUSC3), a potential target gene of miR-UL112-3p, was inversely correlated with miR-UL112-3p expression in GBM tissues and cell lines. Furthermore, we demonstrated that TUSC3 was directly regulated by miR-UL112-3p, and the ectopic expression of TUSC3 reversed the effects of miR-UL112-3p on GBM progression via the AKT signalling pathway. Taken together, these findings collectively demonstrate that miR-UL112-3p exerts its oncogene function by directly targeting TUSC3 in GBM, indicating a potential novel therapeutic target for GBM.

Glioblastoma (GBM) is the most aggressive and lethal brain tumour that is a potential hazard to human health, with a median survival of 12–14 months^1^,^2^. It is characterised by highly invasive behaviour and infiltrative proliferation, which account for its high mortality^3^. Although current treatments like surgery, radiation therapy and chemotherapy are available, recurrence is apparently unavoidable due to its infiltrative growth. Establishing effective methods and creating novel therapies are considered significant to improve GBM poor prognosis.

Human cytomegalovirus (HCMV or HHV5), belonging to the beta subfamily of *Herpesviridae* characterised by linear dsDNA, is ubiquitous and a highly prevalent human pathogen^4^. Following primary infection, the virus establishes a life-long latent infection. During latency, the viral genome is mainly present in myeloid lineage cells and is asymptomatic, but reactivation may cause severe disease in immunocompromised patients^5^. Recently, sufficient evidence has emerged to suggest that HCMV is correlated with the malignant phenotype in GBM, and elements of its biology overlap those considered to be hallmarks of cancer. HCMV proteins and nucleic acid products are expressed in GBM so that HCMV infection is strongly associated with GBM pathogenesis^6^.

MicroRNAs (miRNAs) are short, single-stranded RNA molecules that function in the regulation of gene expression via direct binding to the target mRNAs (including 3′ untranslated regions (3′UTRs), 5′ untranslated regions (5′UTR) and coding region)^7^. They are involved in the regulation of various biological processes, including cell proliferation, differentiation, apoptosis, metastasis, and angiogenesis^8^. Several studies have identified 20–30 mature miRNAs encoded by HCMV^9^,^10^,^11^. *Herpesviridae*-encoded miRNAs have been shown to target various aspects of virus and cell biology, including viral transcriptional activators and immune evasion genes, as well as cellular genes involved in cell cycle regulation, apoptosis, signal transduction and vesicular trafficking^12^,^13^. However, the important role of HCMV-encoded miRNAs in cancer progression, especially GBM, has been rarely reported, and its relevance in GBM clinical pathologies remains uncertain. Moreover, because of the lack of relevant information about HCMV-encoded miRNAs in GBM and other tumours, it seems essential to elaborate its function in tumours to explore diagnostic and therapeutic strategies in patients.

In this study, we first detected the expression of HCMV-encoded miRNAs and found that the expression of miR-UL112-3p was significantly enhanced in HCMV-positive GBM tissue. In addition, we investigated the miR-UL112-3p expression level and its clinicopathological and prognostic significance in GBM patients. Next, we examined the role of miR-UL112-3p in GBM cells *in vitro* and identified the new target TUSC3 of miR-UL112-3p. Our findings demonstrated that HCMV-encoded miR-UL112-3p might act as a tumour regulator by directly targeting TUSC3 in GBM.

## Results

### Clinical significance of miR-UL112-3p expression in glioma

**Figure 1:**
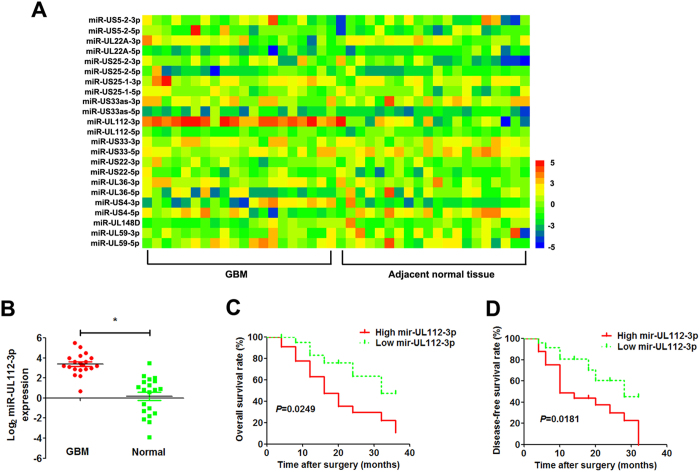
miR-UL112-3p is up-regulated in HCMV-positive GBM. (**A**) Heatmap illustrating miRNome profiles for the 20 HCMV-positive GBM specimens. The log2 values were calculated for each sample by normalising to counting number reads alone. (**B**) Mature miR-UL112-3p expression was determined by qPCR in GBM and adjacent normal tissue. Data are expressed as the mean ± SD. *P < 0.05, n = 20. (**C,D**) Kaplan–Meier analyses of the correlations between the miR-UL113-3p expression level and overall survival rate and disease-free survival rate of GBM patients (n = 20). For each cohort, subgroups were divided according to the mean value of the cohort.

All of the HCMV-positive glioma specimens were confirmed by Immediate Early 86 protein (IE86) immunohistochemistry staining (Supplementary Figure S1). We detected the expression of miR-UL112-3p in 20 pairs of HCMV-positive GBM tissues and matched adjacent normal tissues from GBM patients who received surgery at our hospital, and a heatmap analysis of the HCMV miRNomes was undertaken (Fig. 1A). The results showed that the expression of miR-UL112-3p was significantly up-regulated in GBM tissues compared with adjacent normal tissues (Fig. 1B). The miR-UL112-3p expression levels were classified as either low or high according to the median of the cohort. As shown in Table 1, high expression of miR-UL112-3p was found to significantly correlate with unfavourable variables, including tumour size (*P* = 0.0012), degree of differentiation (*P* = 0.0079), and WHO grade (*P* = 0.0095). However, there were no significant differences observed with age, gender and Karnofsky performance status (KPS). Furthermore, Kaplan–Meier analysis revealed that patients with high miR-UL112-3p expression had significantly lower tumour-free survival and overall survival rates (Fig. 1C,D).

### miR-UL112-3p regulates biological behaviour in GBM cells

**Figure 2:**
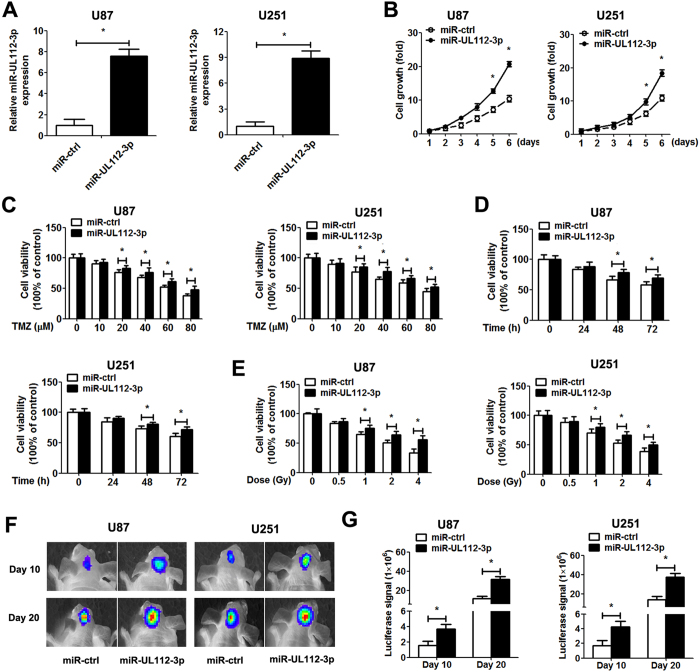
miR-UL112-3p promotes GBM cell growth *in vitro* and *in vivo*. (**A**) The expression of miR-UL112-3p was measured by qPCR analysis. (**B**) The CCK-8 assay was performed to examine cell proliferation at the indicated time points. Absorbance at day 1 was assigned a value of 1. (**C–E**) Cell viability of GBM cells following TMZ and radiation treatments. (**F**) Representative the luciferase signals images of orthotopic implantation from nude mice were shown. (**G**) Quantification of the luciferase signals from the nude mice after 10 and 20 days of implantation. Data are expressed as the mean ± SD. *P < 0.05, n = 5.

To elucidate the role of miR-UL112-3p in GBM cells, we generated stable GBM cell lines (U87 and U251) overexpressing miR-UL112-3p. We also up-regulated and down-regulated the miR-UL112-3p by trasnsfection with miR-UL112-3p mimics/inhibitor in primary GBM cells. The expression of miR-UL112-3p was assessed by qPCR in GBM cells (Fig. 2A and Supplementary Figure S2A). CCK-8 assays showed that miR-UL112-3p overexpression significantly promoted the viability of GBM cells, and this effect increased with time. Down-regulation of miR-UL112-3p revealed an inhibitory effect on GBM cell growth (Fig. 2B and Supplementary Figure S2B). Next, we investigated the effect of miR-UL112-3p combination with temozolomide (TMZ)/radiation treatment on GBM cell viability. Overexpression of miR-UL112-3p enhanced TMZ/radiation resistance of GBM cells, while down-regulation of miR-UL112-3p in GBM cells raised the chemosensitivity/radiosensitivity (Fig. 2C–E and Supplementary Figure S2C–E). Consistent with these *in vitro* data, miR-UL112-3p overexpression significantly accelerated *in vivo* growth of intracranial tumours (Fig. 2F,G).

**Figure 3:**
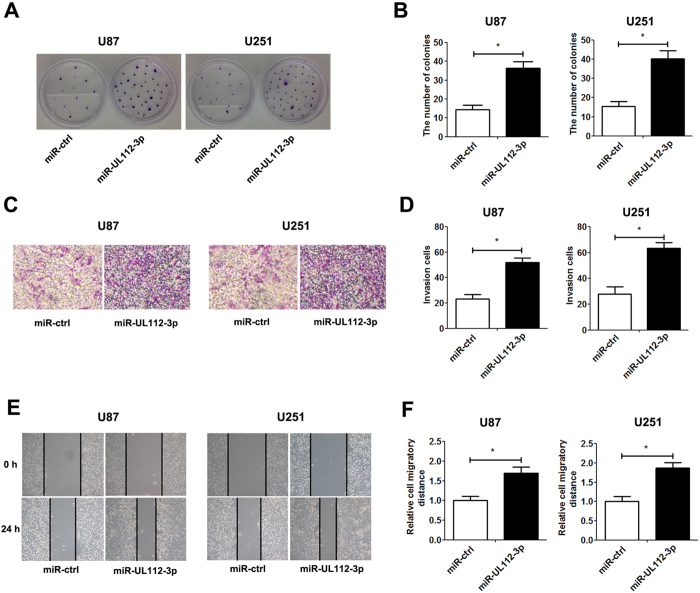
miR-UL112-3p enhances the clone-formation, invasion and migration in GBM cells. (**A**) The representative images of the clone-formation assay. (**B**) Quantification of the colony number of GBM cells after 14 days of incubation. (**C**) The representative images of invasive GBM cells after 24 h of culture in Matrigel invasion chambers. (**D**) Quantification of the number of transmembrane cells. (**E**) Representative images were taken at 0 and 24 h to assess the cell migration into the open space. (**F**) Quantification of the migration distance was achieved by measuring wound closure. The data are expressed as the mean ± SD. *P < 0.05, n = 5.

**Figure 4:**
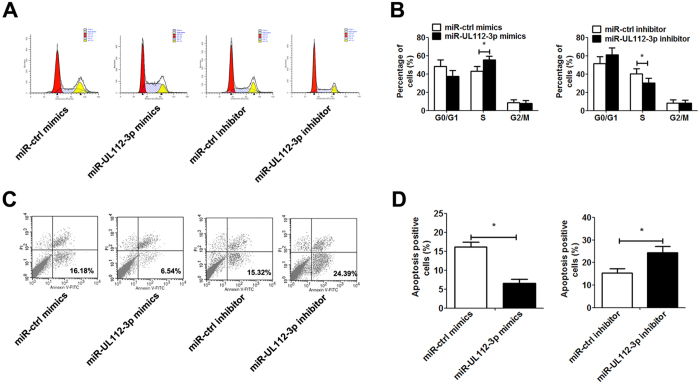
miR-UL112-3p regulates cell cycle and cell apoptosis in primary GBM cells. 48 h after transient transfection with miR-UL112-3p mimics/inhibitor (or control mimics/inhibitor) in primary GBM cells. (**A**) Representative images of the cell cycle. (**B**) Frequencies of cells at different stages of the cell cycle. (**C**) Primary GBM cells were treated with 1 ng/ml TNF-α for 12 h and then underwent apoptosis assay. Representative images of cell apoptosis. (**D**) Rate of cell apoptosis. The data are expressed as the means ± SD. *P < 0.05, n = 5.

In addition, the overexpression of miR-UL112-3p significantly promoted clone-formation, cell migration and invasion, whereas down-regulation of miR-UL112-3p showed an inhibitory effect on clone-formation, cell migration and invasion in GBM cells (Fig. 3 and Supplementary Figure S3). Flow cytometry assays further revealed that transfection with miR-UL112-3p mimics obviously increased the percentage in S phase and reduced TNF-α-induced apoptosis in GBM cells, while transfection with miR-UL112-3p inhibitor exerted the inverse effect (Fig. 4).

### miR-UL112-3p directly inhibits TUSC3 gene expression via targeting its mRNA

**Figure 5:**
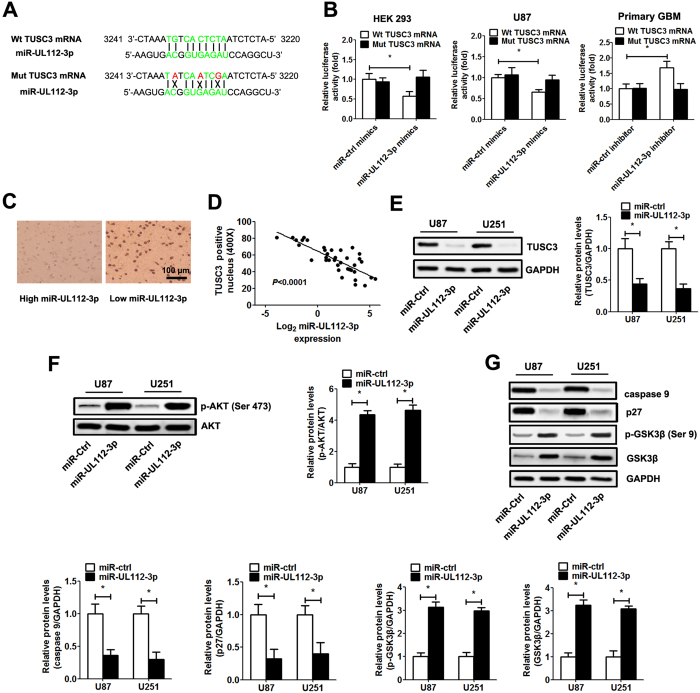
miR-UL112-3p negatively regulates TUSC3 expression in GBM. (**A**) The predicted miR-UL112-3p target sequences in TUSC3 mRNA is shown (green bases indicate matching base pairs, and the red bases represent non-matching base pairs). (**B**) Luciferase assays of HEK293 and GBM cells co-transfected with wild-type/mutant pMIR-REPORT 3′UTR of TUSC3 and miR-UL112-3p mimics/inhibitor or the negative control, as indicated. (**C**) Immunohistochemistry analysis of TUSC3 expression in GBM specimens. (**D**) Spearman’s correlation analysis was used to determine the correlation between the expression levels of TUSC3 and miR-UL112-3p in human GBM samples; Spearman’s correlation, r = −0.7589 (n = 20). (**E**) The expression of TUSC3 was detected by Western blotting in GBM cells. (**F,G**) The protein levels of total and phosphorylated AKT, caspase 9, p27, p-GSK3β and GSK3β in GBM cells, as determined by Western blot analysis. Relative protein expression was calculated based on densitometric analysis of band intensities. Full-length blots are presented in Supplementary Figures S4–S6. The data are expressed as the means ± SD. *P < 0.05, n = 5.

To further evaluate how miR-UL112-3p exerts its function in GBM, bioinformatics (NCBI Nucleotide Blast and lasergene 6.0 software) was used to predict the target gene. As shown in the Fig. 5A, the TUSC3 mRNA harboured a potential miR-UL112-3p binding site (3227–3236 region of TUSC3 mRNA, ATCTCACTGT). To validate whether miR-UL112-3p can directly bind to and regulate the levels of TUSC3 mRNA through the predicted binding sites, we cloned the wild-type and mutant sequence that contain the potential binding site using the luciferase reporter gene system (Fig. 5A). As expected, the miR-UL112-3p mimics led to significantly reduced luciferase activity for the wild-type, whereas the miR-UL112-3p inhibitor increased wild-type luciferase activity. By contrast, the activity of the luciferase reporter gene linked to the mutant sequence did not change in the presence of the miR-UL112-3p mimics/inhibitor (Fig. 5B). We further determined the expression of TUSC3 using immunohistochemistry and Western blotting in specimens and GBM cells. Immunohistochemistry analysis showed that the expression of TUSC3 levels in tumours with low miR-UL112-3p expression was significantly higher than those in tumours with high miR-UL112-3p expression (Fig. 5C). We also found that the expression of miR-UL112-3p showed an inverse correlation with the level of TUSC3 in the GBM samples (Fig. 5D). Western blot analysis demonstrated that TUSC3 expression was significantly down-regulated after the overexpression of miR-UL112-3p in GBM cells (Fig. 5E).

A previous study showed that the up-regulation of TUSC3 has been shown to lead to the inhibition of the AKT pathway^14^. Next, the status of the AKT pathway was examined in the GBM cells in which the TUSC3 expression was altered by the overexpression of miR-UL112-3p. The results indicated that the knockdown of TUSC3 caused by the overexpression of miR-UL112-3p could enhance the activity of the AKT pathway (Fig. 5F), which resulted in elevated p-GSK3β, GSK3β expression and decreased p27, caspase 9 expression (Fig. 5G).

### miR-UL112-3p exerts its biological effects on GBM via TUSC3/AKT axis

**Figure 6:**
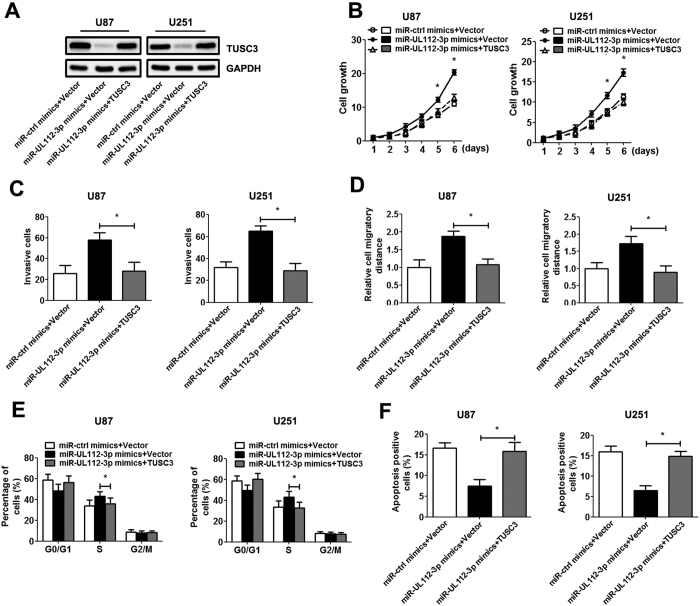
Ectopic expression of TUSC3 inhibits the effect of miR-UL112-3p in GBM cells. U87 and U251 cells were infected with the empty vector or TUSC3 adenovirus (MOI = 50) for 48 h. (**A**) The expression of TUSC3 protein was analysed by Western blotting. Full-length blots are presented in Supplementary Figure S7. (**B**) Cell growth curves for GBM cells were measured by the CCK-8 assay. (**C**) Transwell assays. (**D**) Wound-healing assay. (**E**) Cell cycle progression was analysed by flow cytometry. (**F**) The cell apoptosis rate was analysed by flow cytometry. The data are expressed as the means ± SD. *P < 0.05, n = 5.

To further evaluate the contribution of TUSC3 to the biological effects of miR-UL112-3p, we restored TUSC3 expression in GBM cells by infection with TUSC3 adenovirus (lacking the potential binding site of miR-UL112-3p). Western blot analysis determined the increased expression of TUSC3 protein in the GBM cells after infection (Fig. 6A). Subsequent results showed that the restoration of TUSC3 expression inhibited the effect of miR-UL112-3p on GBM, resulting in a significant decrease in cell growth, invasion and migration (Fig. 6B–D). Similarly, the re-expression of TUSC3 exhibited an apparent decreased percentage of cells in S phase and rescued apoptosis in GBM cells (Fig. 6E,F).

**Figure 7:**
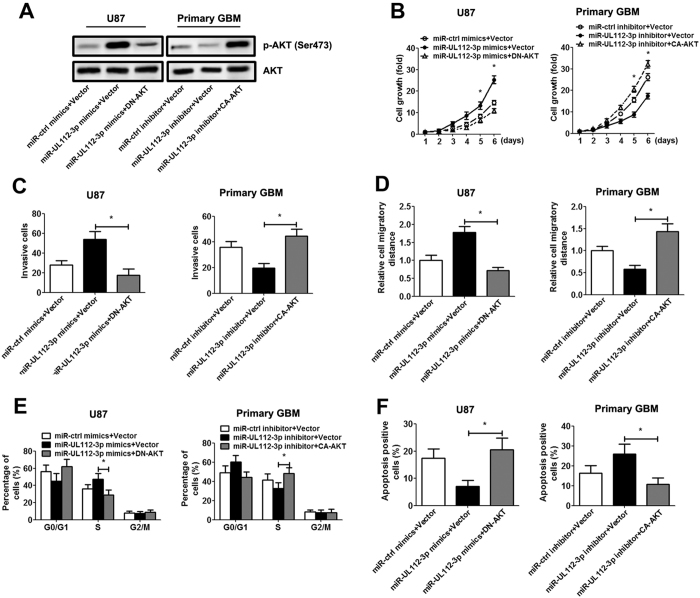
Inactivation or reactivation of AKT signalling reverses the effect of miR-UL112-3p in GBM cells. U87 and primary GBM cells were transfected with the empty vector or DN-AKT/CA-AKT for 48 h. (**A**) The expression of total and phosphorylated AKT potein was analysed by Western blotting. Full-length blots are presented in Supplementary Figure S8. (**B**) Cell growth curves for U87 and primary GBM cells were measured by the CCK-8 assay. (**C**) Transwell assays. (**D**) Wound-healing assay. (**E**) Cell cycle progression was analysed by flow cytometry. (**F**) The cell apoptosis rate was analysed by flow cytometry. The data are expressed as the means ± SD. *P < 0.05, n = 5.

Furthermore, miR-UL112-3p mimics/inhibitor and plasmid expressing constitutively active p-AKT (CA-AKT)/dominant negative p-AKT mutant (DN-AKT) were co-transfected in GBM cells, and the corresponding changes in p-AKT expression were verified by immunoblotting (Fig. 7A). Our result suggested that primary GBM cell transfected with CA-AKT exhibited a significant increase cell growth, invasion, migration, S phase percentage and a decrease cell apoptosis. On the other hand, transfected with DN-AKT blocked miR-UL112-3p-mediated facilitation effect on GBM cells (Fig. 7B–F).

## Discussion

Evidence is accumulating to support a role for infectious agents in the development and progression of human cancer. In addition, an ever increasing percentage of human malignancies (hepatoma, cervical cancer and gastric cancer) in the last several decades have been attributed to virus infection^15^. However, until now, HCMV has not been clearly implicated in human malignancies. Growing evidence that HCMV is specifically detected in various human malignancies at low levels of expression raises the possibility that chronic infection^16^ of HCMV could induce the same type of “smouldering inflammation” seen with other pathogens associated with cancer^17^. It has been hypothesised for over a decade that HCMV might play an “oncomodulatory” role in the neoplastic process, including proliferation, apoptosis, invasion, metabolism and immunity dysfunction.

On the basis of research, there is sufficient evidence to conclude that HCMV sequences and viral gene expression exist in most, if not all, malignant gliomas and that HCMV could modulate the cell biology in GBM by interacting with key signalling pathways^18^. Recently, evidence has been accumulating to support a new perspective that viral mRNA can directly alter host cell physiology^19^. The latest study indicates that cytomegalovirus-encoded CMV-70-3p increases GBM cancer stem cells stemness and acts as a potential regulator of CMV-mediated glioma progression^20^, implying that HCMV-encoded miRNAs are involved in regulating carcinoma pathophysiological processes.

In the current study, we initially detected the expression level of HCMV-encoded in 20 samples of GBM tissues and paired adjacent normal tissues. Our data showed that the expression of miR-UL112-3p was elevated in HCMV-positive GBM tissues, and increased miR-UL112-3p expression was associated with larger tumour size, poorer tumour differentiation and higher WHO grade, suggesting that miR-UL112-3p might be closely related to the tumour development of GBM. Moreover, patients with a high expression of miR-UL112-3p had shorter overall and disease-free survival. We demonstrated that miR-UL112-3p was an independent prognostic marker for predicting the overall and disease-free survival of GBM patients. Altogether, these results indicate that the status of miR-UL112-3p is critical for the prognosis determination in GBM patients. Our results lend credence to a previous study indicating that a higher prevalence of miR-UL112-3p was detected in GBM patients^21^.

miR-UL112-3p has been demonstrated to cooperate in evading the host antiviral immune response by the down-regulation of endogenous TLR2 during infection^22^. Recent evidence has shown that miR-UL112 can directly and broadly affect NK cell cytotoxicity through type I IFN-dependent signalling, perhaps providing another example of how HCMV establishes chronic infection in the presence of fully functional host immune surveillance^23^. It is clear that HCMV utilises miRNAs to regulate their own genes, as well as those of the host cell, during infection to immune evasion^24^,^25^. While targets of HCMV miRNAs are being uncovered, much remains unknown about their functions during infection. In this study, the overexpression of miR-UL112-3p in GBM cells promoted cell growth by increasing cells in S phase and decreasing cell apoptosis, indicating that miR-UL112-3p may be a novel agent and may play a critical role in GBM progression. Our data reveal a novel biological role of HCMV-encoded miRNAs in GBM.

In addition, an inverse correlation between the expression of miR-UL112-3p and TUSC3 protein was observed in GBM tissues and cells. We confirmed that TUSC3 was a direct downstream target of miR-UL112-3p, and it was implicated in the functional effect of miR-UL112-3p in GBM. TUSC3, also known as N33, has been described and identified as a homologue of the yeast Ost3p subunit of the oligosaccharyltransferase (OST) complex^26^. TUSC3 is recognised as a potential tumour suppressor gene. It is located on chromosomal band 8p22, which is frequently deleted in several tumour types, including colorectal cancer^27^, prostate^28^ and pancreatic cancer^29^. Recent studies have provided direct evidence that TUSC3 regulates the proliferation and invasion of GBM cells by inhibiting the activity of the AKT signalling pathway^14^. In this study, we found that the facilitation effect of miR-UL112-3p on GBM cells could be reversed by TUSC3 ectopic expression, suggesting that miR-UL112-3p regulate GBM pathophysiological processes by suppressing TUSC3 expression. This work first implied the critical role of HCMV-encoded miRNA in GBM pathogenesis and provided a novel identified mechanism by viral miRNA in GBM progression.

**Figure 8:**
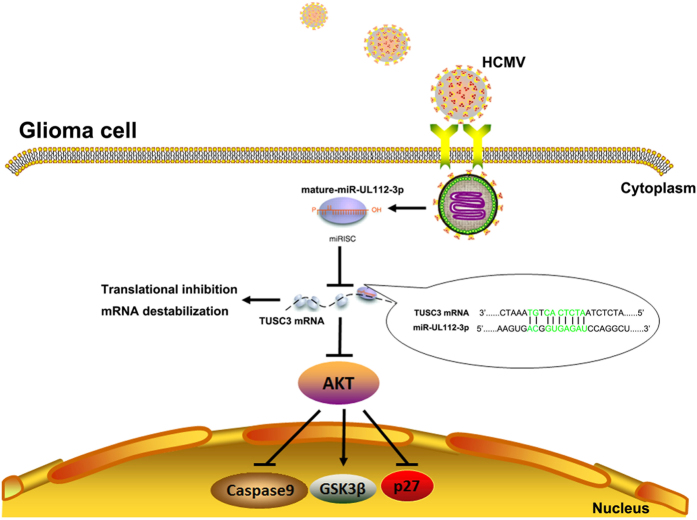
Model illustrating that HCMV-encoded miR-UL112-3p induces GBM progression that is mediated by TUSC3/AKT axis.

In conclusion, we showed that miR-UL112-3p expression was up-regulated in HCMV-positive GBM tissues, and its high expression was correlated with poor prognostic features and lower overall survival rates. Our data revealed the important molecular mechanism by which HCMV-encoded miR-UL112-3p exerts its effects on cell biology in GBM by controlling the host’s protein (Fig. 8). Taken together, this new finding may also be a prognostic marker and therapeutic target for GBM patients.

## Materials and Methods

### Patients and tissue samples

Forty cases of HCMV-positive glioma specimens and matched adjacent normal tissues were obtained from patients who underwent curative resection at the Department of Pathology, No. 401 Hospital of PLA between 2010 and 2013. A portion of the tumour tissue was saved and made into paraffin sections for histological analysis. Additionally, the remaining tissue was snap-frozen in liquid nitrogen and then stored at −80 °C for RNA extraction and other biological molecular experiments. None of the patients were pretreated with interventional or other treatment prior to surgery. The histopathologic diagnoses of these tissue samples were in strict accordance with 2016 World Health Organization (WHO) criteria by two established neuropathologists. The clinical samples were obtained with informed consent, and the study protocol was approved by the Ethics Committee of No. 401 Hospital of PLA. All human-related methods were performed in accordance with the relevant guidelines and regulations.

### RNA extraction and quantitative real-time PCR analysis (qPCR)

qPCR analysis of HCMV miRNAs was carried out as previously described^4^. Total RNA was isolated from human tissues or harvested cells using TRIzol (Invitrogen, CA, USA) according to the manufacturer’s instructions. One nanogram of total RNA was converted into cDNA by reverse transcription with oligo-dT in a single step, using the Reverse Transcription Kit (QIAGEN, Shanghai, China). miRNA expression was quantified by qPCR using the SYBR PCR Master Mix (QIAGEN, Shanghai, China) in the ABI7500 system (Life Technologies, Carlsbad, CA, USA). The relative quantification of miRNAs was calculated using the 2^−ΔΔCt^ method. The data were normalised using U6 as an internal control and were measured relative to a calibrator sample as the external control.

### Cell culture and generation of stable cell lines

GBM primary cultures were derived from patient fresh GBM specimens. Single-cell suspensions were prepared by digesting small pieces (<1 mm) with 0.05% Trypsin-EDTA for 30 min and passing through a cell strainer (70 μm) followed by mechanical dissociation. The isolated cells were resuspended in RPMI 1640 supplemented with 5% foetal bovine serum (FBS, Gibco, Grand Island, NY, USA). The viability of primary cells was assessed by trypan blue exclusion assay (greater than 95%). GBM primary cultures were maintained in DMEM/F12 (Gibco) supplemented with 10% FBS (Gibco) and 100 U/ml penicillin/streptomycin (Gibco). Human GBM cell lines (U87 and U251) and human embryonic kidney (HEK) 293 cells were obtained from Shanghai Institutes for Biological Sciences Cell Resource Centre and were grown in Dulbecco’s modified Eagle’s medium (Invitrogen, Carlsbad, CA, USA) supplemented with 10% FBS and 100 U/ml penicillin/streptomycin. All cultures were grown at 37 °C under an atmosphere of 5% CO_2_ and 95% air.

Stable cell lines were generated as previously described^30^. Briefly, the total length of the miR-UL112-3p sequence was synthesised from GenePharma (Shanghai, China) and was cloned into pMSCV-PIG vectors. U87 and U251 cells were infected with retroviruses generated in phoenix cells. After 72 h, the cells were selected with 2 μg/ml puromycin.

### Cell transfection

The miR-UL112-3p mimics/inhibitor and control miRNA mimics/inhibitor were synthesized from Genepharma (Shanghai, China). DN-Akt, CA-Akt and the empty expression vector were purchased from Upstate Biotechnology (Lake Placid, NY, USA). Transfections were carried out at a final oligonucleotide concentration of 50 nM using Lipofectamine^TM^ 2000 reagent (Invitrogen, Carlsbad, CA, USA) when GBM cells grown to 70% confluence. All of the assays were performed 48 hours after transfection.

### Cell viability

Cell viability was monitored using cell counting kit-8 (CCK-8; Dojindo Laboratories, Kumamoto, Japan). Briefly, 5 × 10^3^ cells/well were plated into 96-well microplates. Next, the absorptions of the cells were measured at different indicated time points (1, 2, 3, 4, 5 and 6 days after seeding into plates) using a Benchmark Microplate Reader (Bio-Rad Laboratories, Hercules, CA, USA) according to the manufacturer’s instructions. To investigate the inhibitory effect of temozolomide and radiation in cells, a series of concentrations of temozolomide (Sigma, St. Louis, MO, USA, 0, 10, 20, 40, 80 μM) or radiation intensity (0, 0.5, 1, 2, 4 Gy) were added to the cells.

### Clone-formation assays

For the clone-formation assay, 500 cells were seeded onto 10-cm dishes and were cultured at 37 °C in a 5% CO_2_ incubator. After incubation for 14 days, the clones were fixed with cold methanol and then were stained with crystal violet. The clone-formation ability was evaluated as the number of colonies.

### Orthotopic implantation assay

For the orthotopic implantation, the indicated GBM cells (1 × 10^4^) transduced with luciferase were stereotactically injected into the right cerebral cortex at a depth of 3.5 mm of nude mice. Mice were monitored by bioluminescent imaging on the 10^th^ and 20^th^ day after injection. All of the animal procedures were conducted in accordance with the guide for the Care and Use of Laboratory Animals of Fudan University and were approved by the Animal Research Committee.

### Cell cycle and apoptosis analysis

Cell cycle progression and cell apoptosis were performed using a Becton Dickinson FACS/Calibur cytometer (Becton Dickinson Biosciences, Inc., NJ, USA) after 48 h of incubation. For cell cycle analysis, the GBM cells were fixed in 70% cold ethanol followed by incubation with 2 μg/ml bovine pancreatic RNase A (Sigma, St. Louis, MO, USA) and 20 μg/ml propidium iodide (Sigma). Cell apoptosis was detected using the Annexin-V fluorescein isothiocyanate (FITC) Apoptosis Kit (Calbiochem, San Diego, CA, USA) according to the manufacturer’s protocol.

### Transwell invasion and wound-healing assay

For the cell-invasion assay, 1 × 10^5^ cells were added to the upper well of Matrigel-coated invasion chambers (8-μm pore size; Corning Inc., Corning, NY, USA) for 24 h. Next, the cells on the upper side of the membrane were wiped off, and the cells on the lower side of the membrane were fixed in cold methanol and were stained with 0.2% crystal violet (Sigma, St. Louis, MO, USA). The invasive cells were counted under 5 high-power fields in each chamber. An artificial wound was introduced into monolayer cells using a sterile 200-μl pipette tip. The wound closure was monitored and photographed at 0 and 24 hours. The migration ability was evaluated as the distance of wound closure in 5 high-power fields.

### Immunohistochemistry assay

The specimens were fixed in 4% paraformaldehyde at 4 °C for 48 h and then were embedded in paraffin. The sections (5-μm thick) were dewaxed and rehydrated in graded alcohol. Endogenous peroxidase activity was blocked by incubation with 0.3% hydrogen peroxide for 30 min. Subsequently, slide antigen retrieval was accomplished by steam heating for 10 min. The primary TUSC3 monoclonal antibodies (Santa Cruz, CA, USA) and HCMV IE86 (Virostat, Westbrook, ME, USA) were diluted to 1:50 and were incubated with the sections at 4 °C in a humid chamber overnight. After reaction with a biotinylated goat anti-rabbit secondary antibody (Santa Cruz) for 1 h at room temperature, the antigen-antibody reaction was visualised with diaminobenzidine serving as the chromogen.

### Western blot assays

The cells were lysed on ice in RIPA buffer supplemented with protease inhibitors. The protein concentration was determined using the BCA Kit (Pierce, IL, USA), and aliquots of protein were separated by SDS-polyacrylamide gel electrophoresis (PAGE) and transferred onto PVDF membranes. The blots were then probed with antibodies against the primary antibodies, and then with peroxidase-conjugated secondary antibody (Santa Cruz, CA, USA). The bands were visualised with the ECL Detection System (Merck Millipore, Germany). Primary antibodies anti-TUSC3, anti-phospho-AKT (Ser 473), anti-AKT, anti-caspase 9, anti-GSK3β, anti-p-GSK3β (Ser 9), anti-p27 and anti-GAPDH were from Santa Cruz Biotechnology (Santa Cruz, CA, USA).

### TUSC3 adenovirus construction and infection

The TUSC3 adenovirus was constructed using the Ad-easy system as previously described^31^. The TUSC3 and control adenovirus were infected into GBM cells at a density of 50–70% confluency with an MOI of 50. The cells were collected for further analyses performed 48 h after infection.

### Luciferase report assays

The full length mRNA sequence of TUSC3 (3888 bp, NCBI Reference Sequence: NM_006765.3) was synthesised and then inserted into the HindΙΙΙ and MluΙ restriction sites downstream of the luciferase open reading frame of the pMIR-REPORT luciferase vector (Ambion, Carlsbad, CA, USA). For sequence point mutation, site-directed mutagenesis of the potential target site in the TUSC3 mRNA was performed using the QuikChange Site-Directed Mutagenesis kit (Promega, Madison, WI, USA). The correct clones were confirmed by sequencing. HEK 293 and GBM cells were seeded into a 24-well plate (5 × 10^4^ per well) and co-transfected with miR-UL112-3p mimics/inhibitor (50 nM) and the wild-type/mutant pMIR-REPORT luciferase vector (200 ng) using Lipofectamine^TM^ 2000 reagent according to the manufacturer’s instructions. Luciferase activity was detected 48 h after co-transfection using the dual luciferase system kit (Promega, Madison, WI, USA). The relative luciferase activity was calculated by normalising firefly luminescence to that of renilla.

### Statistical analysis

The results were presented as the mean ± SD, and values analyses were carried out using SPSS 17.0 software (SPSS, Inc., Chicago, IL, USA). Heatmap analysis was performed by HemI Version 1.0 software (GPS, Wuhan, China). The statistical significances between two groups were analysed using Student’s t-test. Count data were analysed by Fisher’s exact test. Univariate survival analysis was performed using the Kaplan–Meier method and the log-rank test. Differences were considered statistically significant at *P* < 0.05.

## Additional Information

**How to cite this article:** Liang, Q. *et al*. HCMV-encoded miR-UL112-3p promotes glioblastoma progression via tumour suppressor candidate 3. *Sci. Rep.* **7**, 44705; doi: 10.1038/srep44705 (2017).

**Publisher's note:** Springer Nature remains neutral with regard to jurisdictional claims in published maps and institutional affiliations.

## Supplementary Material

Supplementary Figures

## Figures and Tables

**Table 1:** Correlations between miR-UL112-3p expression in glioma and clinical characteristics.

Group	NO.	Relative miR-UL112-3p expression		*P* value
		Low	High	
Age				
≦50	18	8	10	0.7512
>50	22	12	10	
Gender				
Male	24	10	14	0.3332
Female	16	10	6	
Size of diameter (cm)				
≦3	21	16	5	0.0012*
>3	19	4	15	
Differentiation				
Well and intermediate	25	17	8	0.0079*
Poor	15	3	12	
WHO grade				
I–II stage	17	13	4	0.0095*
III–IV stage	23	7	16	
KPS				
≦90	7	3	4	1.0000
>90	33	17	16	

WHO, World Health Organization; KPS, Karnofsky performance status. *P < 0.05.

## References

[b1] Yang C. . Identification of seven serum microRNAs from a genome-wide serum microRNA expression profile as potential noninvasive biomarkers for malignant astrocytomas. Int J Cancer 132, 116–127, doi: 10.1002/ijc.27657 (2013). 22674182

[b2] Lee H. K. . Mesenchymal stem cells deliver synthetic microRNA mimics to glioma cells and glioma stem cells and inhibit their cell migration and self-renewal. Oncotarget 4, 346–361, doi: 10.18632/oncotarget.868 (2013). 23548312 PMC3712579

[b3] Zhou F. . MicroRNA-300 inhibited glioblastoma progression through ROCK1. Oncotarget, doi: 10.18632/oncotarget.9068 (2016). PMC5095018 27145462

[b4] Meshesha M. K. . The microRNA Transcriptome of Human Cytomegalovirus (HCMV). Open Virol J 6, 38–48, doi: 10.2174/TOVJ-6-38 (2012). 22715351 PMC3377890

[b5] Stark T. J., Arnold J. D., Spector D. H. & Yeo G. W. High-resolution profiling and analysis of viral and host small RNAs during human cytomegalovirus infection. J Virol 86, 226–235, doi: 10.1128/JVI.05903-11 (2012). 22013051 PMC3255895

[b6] Dziurzynski K. . Consensus on the role of human cytomegalovirus in glioblastoma. Neuro Oncol 14, 246–255, doi: 10.1093/neuonc/nor227 (2012). 22319219 PMC3280809

[b7] Bushati N. & Cohen S. M. microRNA functions. Annu Rev Cell Dev Biol 23, 175–205, doi: 10.1146/annurev.cellbio.23.090506.123406 (2007). 17506695

[b8] Kloosterman W. P. & Plasterk R. H. The diverse functions of microRNAs in animal development and disease. Dev Cell 11, 441–450, doi: 10.1016/j.devcel.2006.09.009 (2006). 17011485

[b9] Hook L. . Cytomegalovirus microRNAs. Current opinion in virology 7, 40–46, doi: 10.1016/j.coviro.2014.03.015 (2014). 24769092 PMC4149926

[b10] Dolken L., Pfeffer S. & Koszinowski U. H. Cytomegalovirus microRNAs. Virus genes 38, 355–364, doi: 10.1007/s11262-009-0347-0 (2009). 19291384

[b11] Fannin Rider P. J., Dunn W., Yang E. & Liu F. Human cytomegalovirus microRNAs. Current topics in microbiology and immunology 325, 21–39 (2008). 18637498 10.1007/978-3-540-77349-8_2

[b12] Grey F. . Identification and characterization of human cytomegalovirus-encoded microRNAs. J Virol 79, 12095–12099, doi: 10.1128/JVI.79.18.12095-12099.2005 (2005). 16140786 PMC1212634

[b13] Stern-Ginossar N. . Host immune system gene targeting by a viral miRNA. Science 317, 376–381, doi: 10.1126/science.1140956 (2007). 17641203 PMC4283197

[b14] Jiang Z. . TUSC3 suppresses glioblastoma development by inhibiting Akt signaling. Tumour Biol, doi: 10.1007/s13277-016-5072-4 (2016). 27177902

[b15] Demaria S. . Cancer and inflammation: promise for biologic therapy. J Immunother 33, 335–351, doi: 10.1097/CJI.0b013e3181d32e74 (2010). 20386472 PMC2941912

[b16] Cinatl J. Jr. . Modulatory effects of human cytomegalovirus infection on malignant properties of cancer cells. Intervirology 39, 259–269 (1996). 9078467 10.1159/000150527

[b17] Soderberg-Naucler C. HCMV microinfections in inflammatory diseases and cancer. J Clin Virol 41, 218–223, doi: 10.1016/j.jcv.2007.11.009 (2008). 18164235

[b18] Cinatl J. Jr., Vogel J. U., Kotchetkov R. & Wilhelm Doerr H. Oncomodulatory signals by regulatory proteins encoded by human cytomegalovirus: a novel role for viral infection in tumor progression. FEMS Microbiol Rev 28, 59–77, doi: 10.1016/j.femsre.2003.07.005 (2004). 14975530

[b19] Soroceanu L. & Cobbs C. S. Is HCMV a tumor promoter? Virus Res 157, 193–203, doi: 10.1016/j.virusres.2010.10.026 (2011). 21036194 PMC3082728

[b20] Ulasov I. V. . CMV70-3P miRNA contributes to the CMV mediated glioma stemness and represents a target for glioma experimental therapy. Oncotarget, doi: 10.18632/oncotarget.11175 (2016). PMC5432232 27517625

[b21] Mohammad A. A. . Detection of circulating hcmv-miR-UL112-3p in patients with glioblastoma, rheumatoid arthritis, diabetes mellitus and healthy controls. PLoS One 9, e113740, doi: 10.1371/journal.pone.0113740 (2014). 25462570 PMC4252052

[b22] Landais I. . Human Cytomegalovirus miR-UL112-3p Targets TLR2 and Modulates the TLR2/IRAK1/NFkappaB Signaling Pathway. PLoS Pathog 11, e1004881, doi: 10.1371/journal.ppat.1004881 (2015). 25955717 PMC4425655

[b23] Huang Y. . Hcmv-miR-UL112 attenuates NK cell activity by inhibition type I interferon secretion. Immunol Lett 163, 151–156, doi: 10.1016/j.imlet.2014.12.003 (2015). 25530545

[b24] Dhuruvasan K., Sivasubramanian G. & Pellett P. E. Roles of host and viral microRNAs in human cytomegalovirus biology. Virus Res 157, 180–192, doi: 10.1016/j.virusres.2010.10.011 (2011). 20969901 PMC3383804

[b25] Piedade D. & Azevedo-Pereira J. M. The Role of microRNAs in the Pathogenesis of Herpesvirus Infection. Viruses 8, doi: 10.3390/v8060156 (2016). PMC4926176 27271654

[b26] Schulz B. L. . Oxidoreductase activity of oligosaccharyltransferase subunits Ost3p and Ost6p defines site-specific glycosylation efficiency. Proc Natl Acad Sci USA 106, 11061–11066, doi: 10.1073/pnas.0812515106 (2009). 19549845 PMC2708779

[b27] Gu Y. . TUSC3 promotes colorectal cancer progression and epithelial-mesenchymal transition (EMT) through WNT/beta-catenin and MAPK signalling. J Pathol 239, 60–71, doi: 10.1002/path.4697 (2016). 27071482

[b28] Horak P. . TUSC3 loss alters the ER stress response and accelerates prostate cancer growth *in vivo*. Sci Rep 4, 3739, doi: 10.1038/srep03739 (2014). 24435307 PMC3894551

[b29] Fan X. . Decreased TUSC3 Promotes Pancreatic Cancer Proliferation, Invasion and Metastasis. PLoS One 11, e0149028, doi: 10.1371/journal.pone.0149028 (2016). 26871953 PMC4752499

[b30] Wang K. . Rab17 inhibits the tumourigenic properties of hepatocellular carcinomas via the Erk pathway. Tumour Biol 36, 5815–5824, doi: 10.1007/s13277-015-3251-3 (2015). 25707355

[b31] Xu H., Li G., Yue Z. & Li C. HCV core protein-induced upregulation of microRNA-196a promotes aberrant proliferation in hepatocellular carcinoma by targeting FOXO1. Mol Med Rep 13, 5223–5229, doi: 10.3892/mmr.2016.5159 (2016). 27108614

