# Supplementary material for: HCMV-encoded miR-UL112-3p promotes glioblastoma progression via tumour suppressor candidate 3
Source: Sci Rep. 2017 Mar 17;7:44705. doi: 10.1038/srep44705 (PMC5356197; doi:10.1038/srep44705)
Supplement: Supplementary Figures [file srep44705-s1.pdf]

HCMV-encoded miR-UL112-3p promotes glioblastoma progression via tumour suppressor candidate 3

Qing Liang [1], Kejia Wang [2], Qiliang Cai [1, *], Bin Wang [2, *]

[1] MOE&MOH Key Laboratory of Medical Molecular Virology, School of Basic Medical Sciences, Shanghai Medical College, Fudan University, Shanghai 200032, China

[2] Department of Special Medicine, Qingdao University, Qingdao 266071, Shandong, China

[*] Correspondence to: Qi-Liang Cai, E-mail: qiliang@fudan.edu.cn

Bin Wang, E-mail: qdwangbin1962@163.com

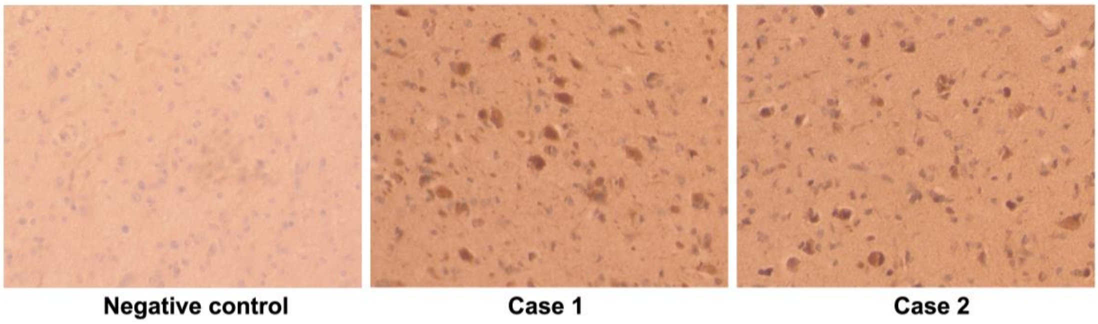

Figure S1. IE86 expression was verified by immunohistochemistry in HCMV-positive GBM specimens.

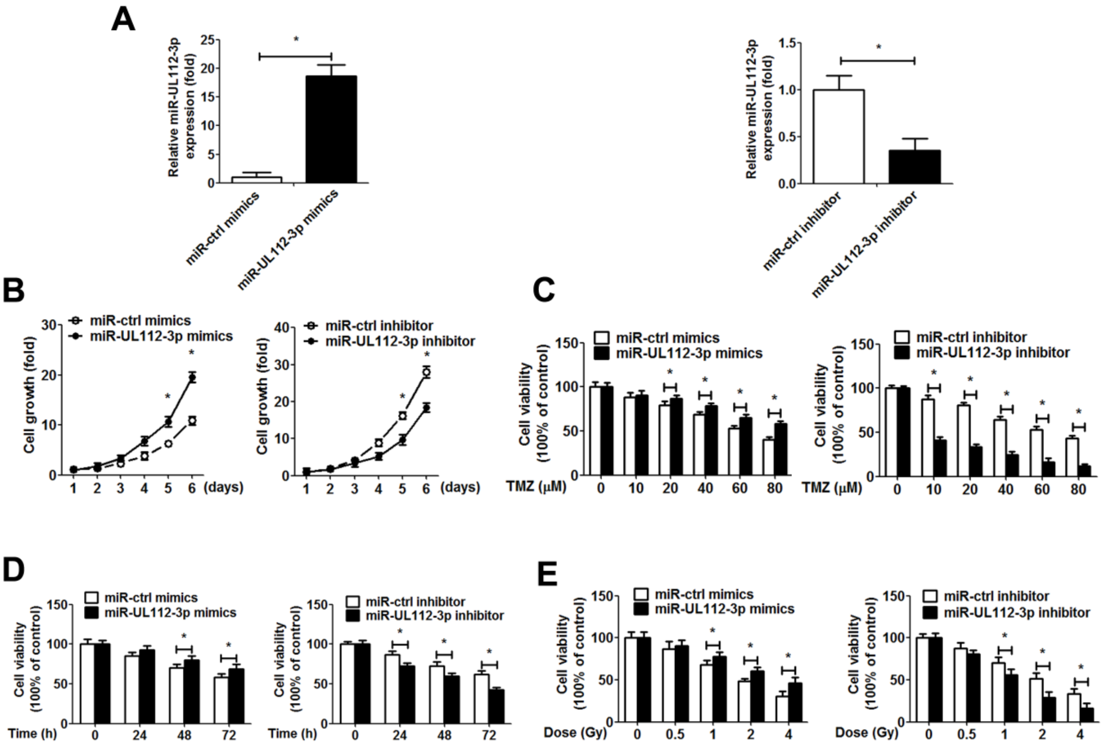

Figure S2. miR-UL112-3p regulates primary GBM cell growth in vitro. Primary GBM cells were transfected with the miR-UL112-3p mimics/inhibitor for 48 h. **(A)**. The expression of miR-UL112-3p was measured by qPCR analysis. **(B)**. The CCK-8 assay was performed to examine cell proliferation at the indicated time points. (**C-E**). Cell viability of GBM cells upon TMZ and radiation treatments. Data are expressed as the mean ± SD. *, $P<0.05$, n = 5.

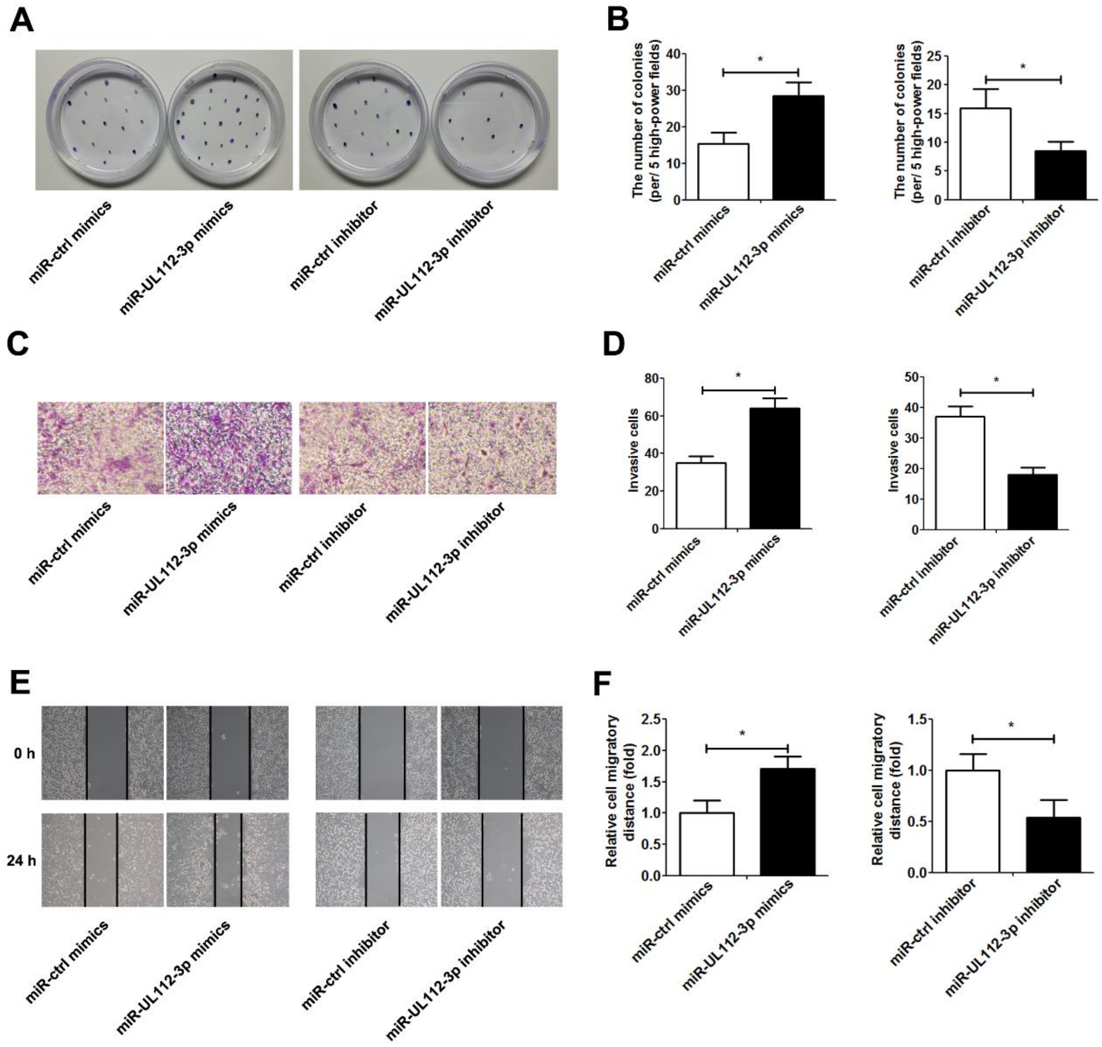

Figure S3. miR-UL112-3p modulates the clone-formation, invasion and migration in primary GBM cells. **(A)**. The representative images of the clone-formation assay. (**B)**. Quantification of the colony number of GBM cells after 14 days of incubation. **(C)**. The representative images of invasive GBM cells after 24 h of culture in Matrigel invasion chambers. (**D)**. Quantification of the number of transmembrane cells. **(E)**. Representative images were taken at 0 and 24 h to assess the cell migration into the open space. (**F)**. Quantification of the migration distance was achieved by measuring wound closure. The data are expressed as the mean ±SD. *, P<0.05, n = 5.

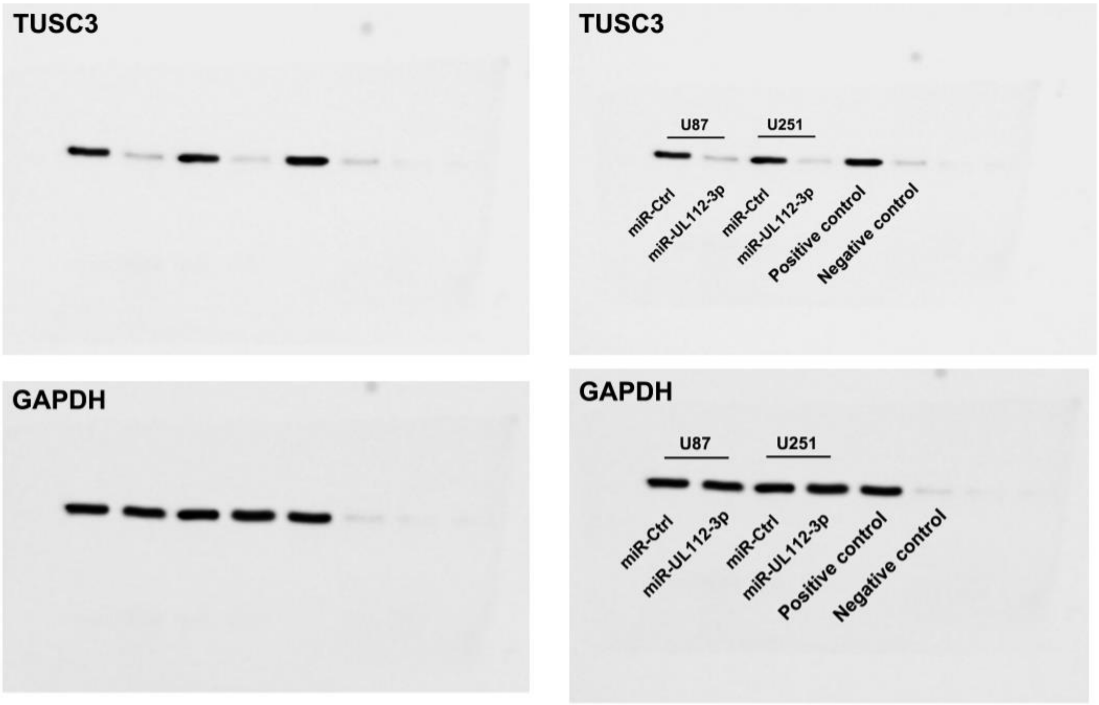

Figure S4. The Full-length immunblots as shown in Figure 5E.

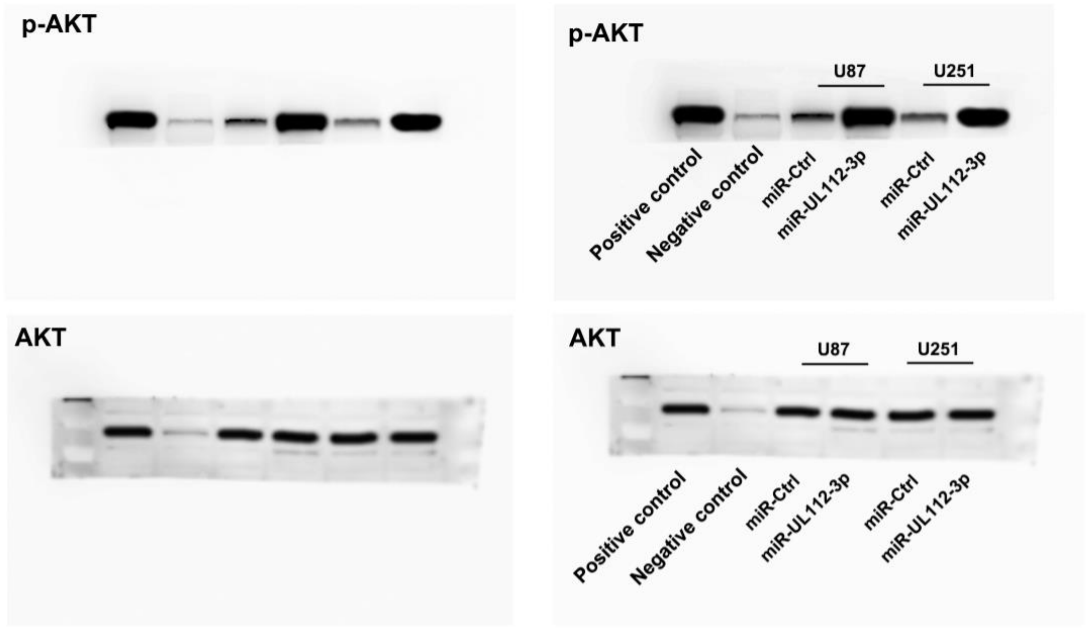

Figure S5. The Full-length immunblots as shown in Figure 5F.

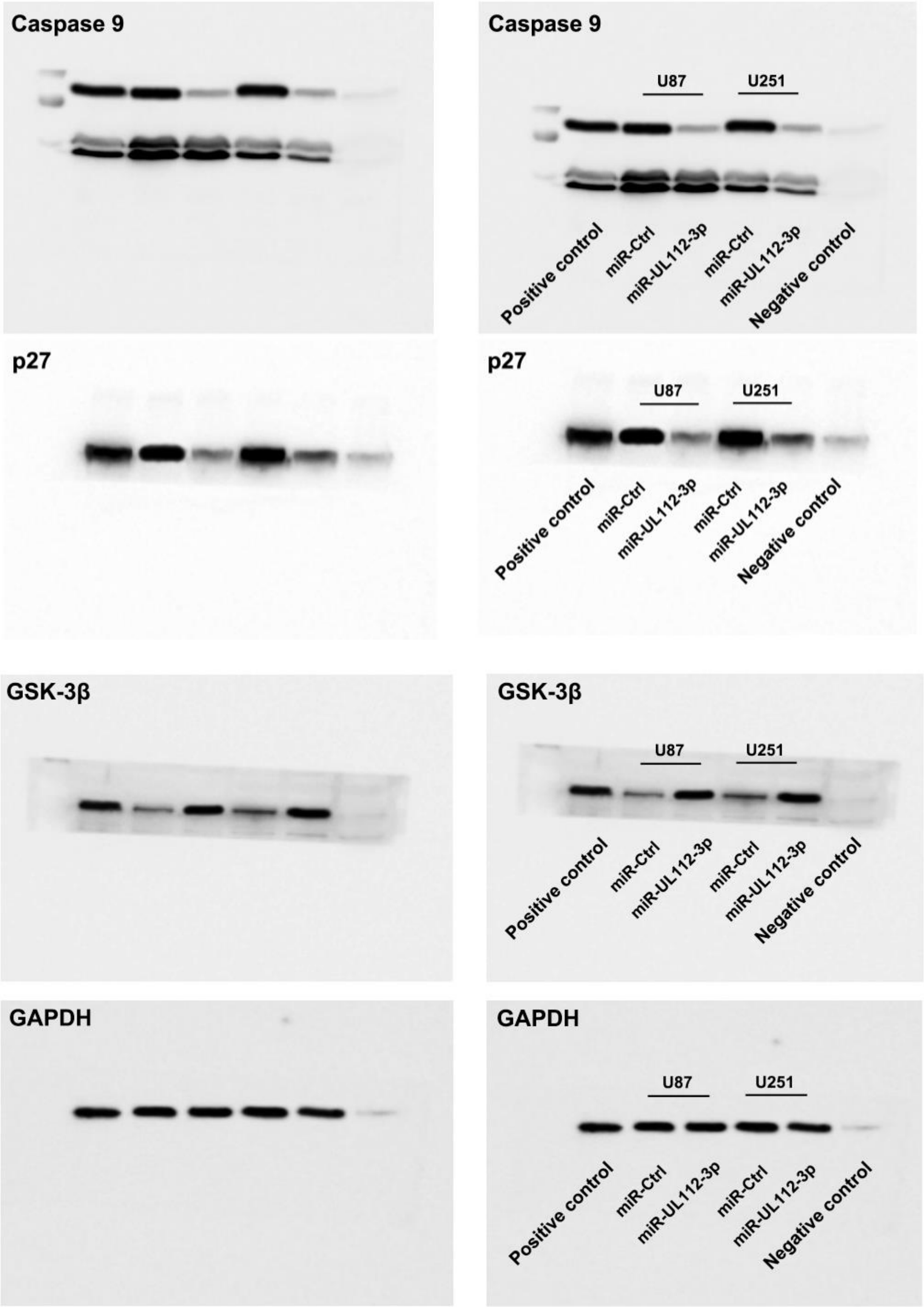
Caspase 9
Caspase 9
U87
U251
Positive control
miR-Ctrl
miR-UL112-3p
miR-Ctrl
miR-UL112-3p
Negative control
p27
p27
U87
U251
Positive control
miR-Ctrl
miR-UL112-3p
miR-Ctrl
miR-UL112-3p
Negative control
GSK-3β
GSK-3β
U87
U251
Positive control
miR-Ctrl
miR-UL112-3p
miR-Ctrl
miR-UL112-3p
Negative control
GAPDH
GAPDH
U87
U251
Positive control
miR-Ctrl
miR-UL112-3p
miR-Ctrl
miR-UL112-3p
Negative control

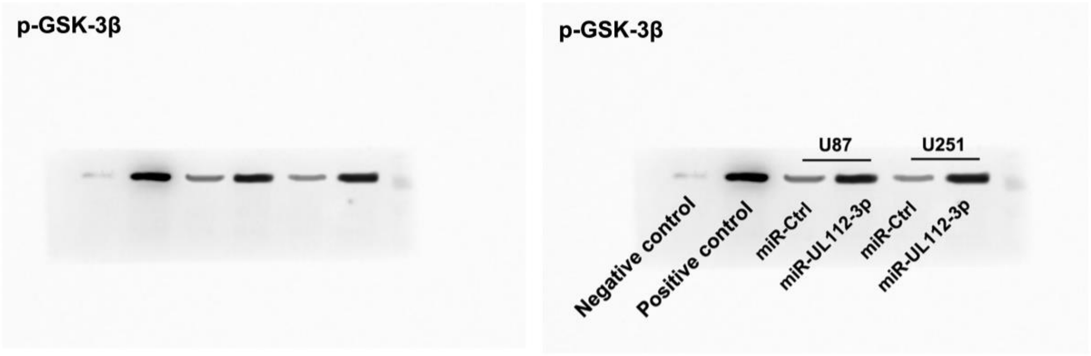

Figure S6. The Full-length immunblots as shown in Figure 5F.

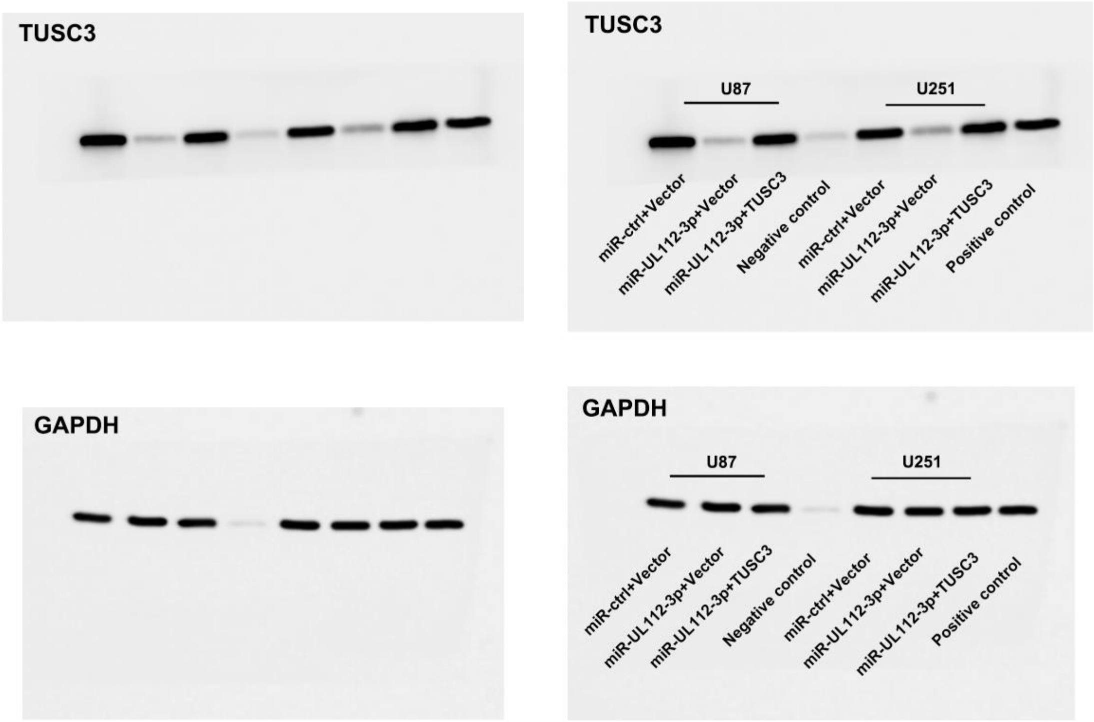

Figure S7. The Full-length immunblots as shown in Figure 6A.

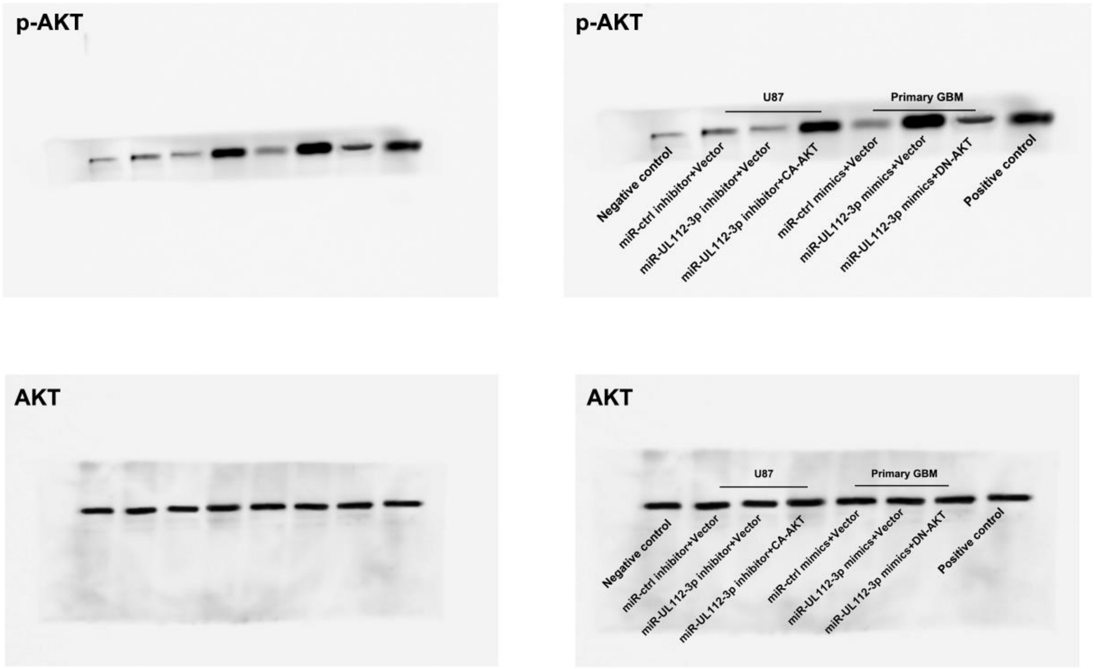

Figure S8. The Full-length immunblots as shown in Figure 7A.
